# Mimicking a Psychiatric Disorder: Heroin-Induced Leukoencephalopathy

**DOI:** 10.7759/cureus.10805

**Published:** 2020-10-05

**Authors:** Mustafa Al-Chalabi, Sohaib Lateef, Khaled Gharaibeh, Purvi Saraiya, Malik Ghannam

**Affiliations:** 1 Department of Neurology , University of Toledo, Toledo, USA; 2 Department of Neurology, University of Toledo, Toledo, USA; 3 Department of Neurology, University of Minnesota, Minneapolis, USA

**Keywords:** toxic leukoencephalopathy, heroin abuse, chasing the dragon

## Abstract

Toxic leukoencephalopathy is a rare cause of encephalopathy. We describe two cases of toxic leukoencephalopathy associated with opiate abuse where they were misdiagnosed and admitted to the psychiatric ward. In our case series, both patients presented with behavioral changes, restlessness, pressured speech, and cognitive impairment for which they were initially labeled as psychiatric patients and were treated as such. However, after an extensive workup to elucidate the etiology of alteration in mental status, toxic leukoencephalopathy associated with heroin abuse was found to be the culprit in both cases. Toxic leukoencephalopathy is a rare condition that can be caused by inhalation of heroin. Clinically, it may present with confusion, behavioral changes, extrapyramidal symptoms, generalized motor deficit, unresponsiveness and even death. Our cases highlight the importance of recognizing the psychiatric presentation of toxic leukoencephalopathy.

## Introduction

Toxic leukoencephalopathy is a broad term that refers to the damage of the white matter caused by exposure to a variety of agents, including drugs of abuse such as heroin as well as irradiation, chemotherapy, and environmental toxins [1-4]. The inhalation of heroin vapor, historically known as “chasing the dragon”, has been associated with toxic leukoencephalopathy in many reported cases [2]. Chasing the dragon refers to a method of heroin inhalation performed by heating up heroin on aluminum foil and inhaling its vapor [2-4]. The practice emerged in Southeast Asia, particularly in Shanghai in the 1920s, extending to Eastern Asia and the United States in the subsequent decades [3-5]. The extent of the white matter damage caused by heroin inhalation correlates to the severity of the neurological deficits. Therefore, the neurological manifestations of toxic leukoencephalopathy can have a broad spectrum of severity ranging from milder syndromes characterized by confusion, inattentiveness, ataxia, and psychomotor symptoms to more severely presenting syndromes characterized by stupor, dementia, abulia, and coma [1].

We present two cases of middle-aged women who came to the emergency department with a history of two to four weeks of bizarre behaviors, inattentiveness, disorientation, and speech abnormalities. While the initial impression was a psychiatric illness in both cases, further investigation revealed heroin inhalation and extensive neurological workup, including brain MRI showed restricted diffusion of the white matter of both cerebral hemispheres with corresponding T2 and fluid-attenuated inversion recovery (FLAIR) hyperintensity consistent with toxic leukoencephalopathy. 

## Case presentation

Case 1

A 51-year-old female with a free past medical history presented to the emergency department (ED) with a two-week history of bizarre behaviors, including grabbing things inappropriately as well as lack of self-hygiene, confusion, inattentiveness at work, and change of speech. Her speech changes were described as delayed and tangential. The family was called to pick up the patient from work on the day of onset due to the inability to work. The family described her as “spaced out” with unsteady gait and stumbling. Over the next several days, the patient stopped going to her work and exhibited behavioral changes such as an inability to dress herself and trying to put her underwear on her head. At one point, she was witnessed walking around the house naked after getting out of the shower and left the water running. She was reported not to shower or brush her teeth for several days, which is a significant change for her, according to the family. 

On examination, the patient was afebrile with blood pressure 150/90 mm/hg, heart rate 110 beats/minute, respiratory rate 16 breaths/minute, and oxygen saturation of 100% on room air. Neurologic examination was remarkable for the inability to answer questions appropriately, inability to name objects, facial dyskinesia, right-sided lower extremity clonus, and diffuse hyperreflexia. Head computed tomography (CT) was unrevealing. Head and neck computed tomography angiogram (CTA) did not demonstrate any vascular abnormalities. Complete blood count (CBC) and basal metabolic panel (BMP) were both unremarkable. Serum glucose was 159 mg/dL, ethanol level was less than 0.01 g/dL, and liver enzymes and ammonia (LFTs) were within normal limits. Vitamin B12 was 389 pg/mL, thyroid profile was normal, and sepsis workup was negative. A urine drug screen was positive for marijuana and opiates. 

**Figure 1:**
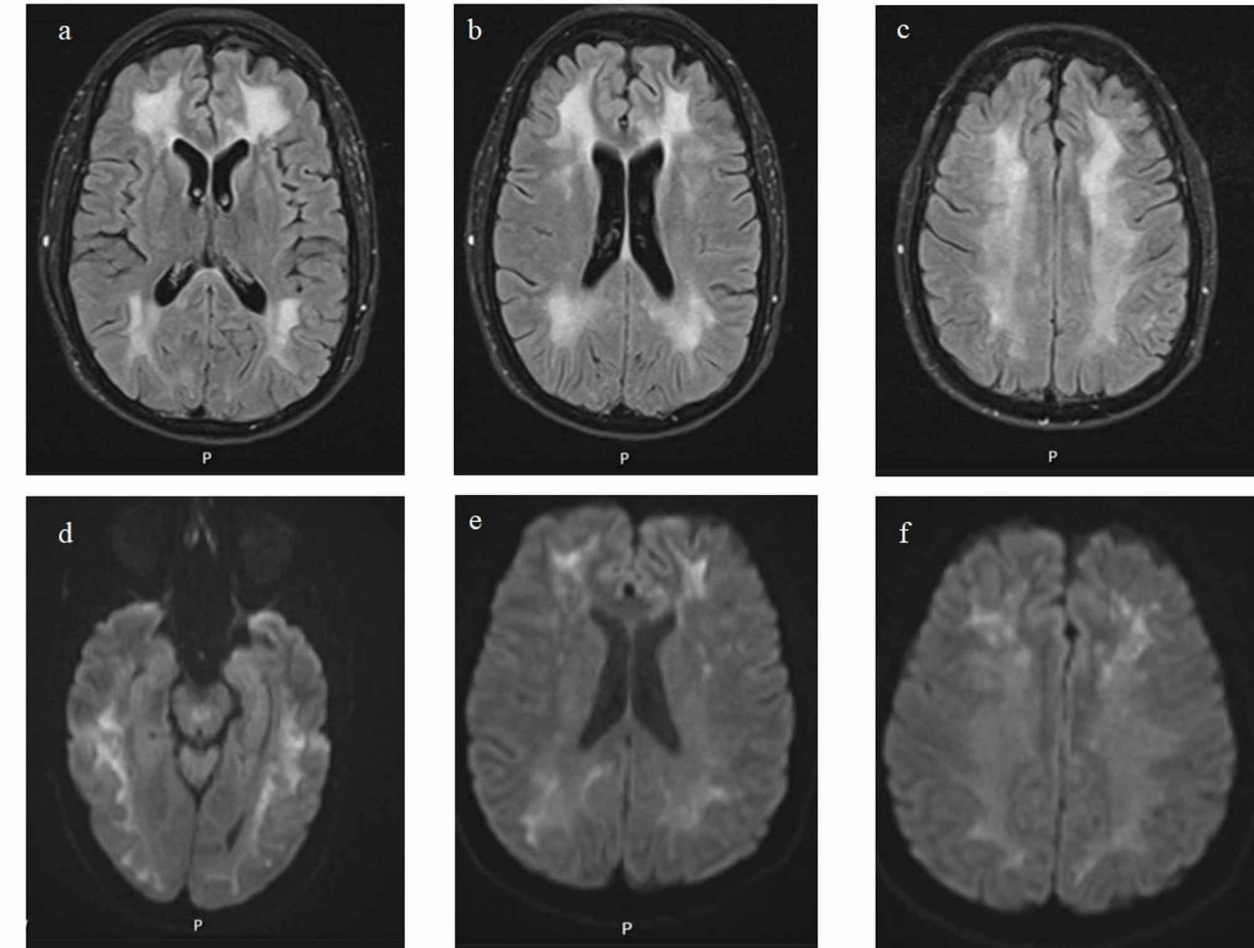
Brain MRI images demonstrate diffuse cortical and subcortical white matter diffusion restriction with corresponding FLAIR hyperintensities of the bilateral cerebral hemispheres A-C; DWI sequences. D-F: T2-FLAIR sequence. FLAIR - fluid-attenuated inversion recovery; DWI - diffusion-weighted imaging

The patient was admitted to the psychiatric ward for acute psychosis. Upon taking more history, the patient was undergoing tremendous stress causing her to use excessive amounts of alcohol and heroin. Moreover, the patient was reported to live in an area where there is a high use of heroin. Brain magnetic resonance imaging (MRI) with and without contrast was performed as part of the evaluation of new-onset psychosis, which revealed restricted diffusion in the white matter of both cerebral hemispheres with corresponding T2 and FLAIR hyperintensity without contrast enhancement (Figure 1). Hence neurology was consulted. Cerebrospinal fluid (CSF) analysis revealed mildly elevated protein at 39 mg/dl, glucose 45 mg/dL, white blood cell count 0-5 cells/µL, and unrevealing CSF cultures. CSF and serum oligoclonal antibodies were within normal limits. Meningitis and autoimmune encephalitis panels were negative. The Creutzfeldt-Jakob disease panel, HIV panel, and syphilis panel were all negative. Extractable nuclear antigen (ENA) panels including anti-Jo, anti-scleroderma scl-70, anti-RNP, anti-Smith, anti-ro and anti-la were all negative. Considering the brain MRI findings and the evidence of heroin use, toxic leukoencephalopathy was the default diagnosis after excluding all the other etiologies. The patient had supportive treatment during her hospital stay consisting of vitamin C, vitamin E, and coenzyme Q, as well as anxiolytics and antipsychotics. On discharge, the patient remained non-communicative, but facial dyskinesia resolved. The patient was discharged to a skilled nursing facility. The patient failed to follow up after discharge.

Case 2

**Figure 2:**
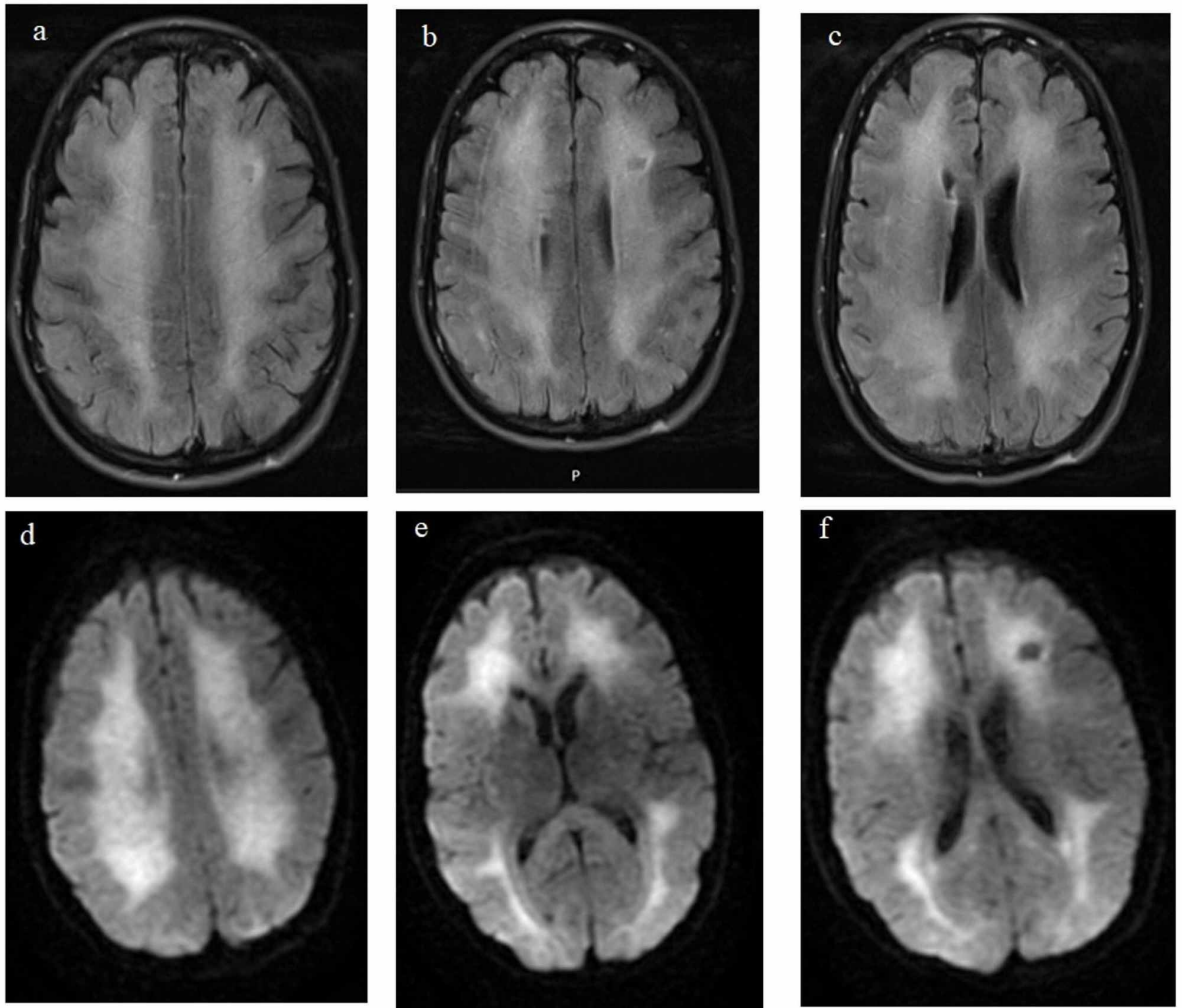
MRI images of the brain showing diffuse cortical and subcortical white matter FLAIR hyperintensity and diffusion restriction. A-C: FLAIR sequence. D-F: DWI sequence. DWI - diffusion-weighted imaging; FLAIR - fluid-attenuated inversion recovery

A 42-year-old female presented with behavioral change, including misplacing personal items, getting lost while driving, and occasional difficulty of short-term memory and visual hallucination. The patient was brought by her family to the ED. The patient had a remarkable history of heroin abuse and was previously placed on Suboxone®. She was admitted to a psychiatric ward with the diagnosis of conversion disorder. However, while in the psychiatric ward, she started exhibiting progressive ambulation, speech difficulties, and kinetic postural tremors. These changes prompted a brain MRI which showed extensive white matter disease concerning for leukoencephalopathy (Figure 2). She was therefore transferred to the neurology general inpatient service for further management. 

On examination, the patient was afebrile with blood pressure 134/78, pulse 96 beats/minute, and oxygen saturation 96% on room air. Her neurological exam was significant for inability to track, inability to verbalize, lack of comprehension, significant paratonia, triple flexion bilaterally, and crossed adductor sign. Labs including complete blood count (CBC), basic metabolic panel (BMP), liver function test (LFT), serum glucose level, sepsis workup, autoimmune panel, heavy metal screen, myoglobin, lactate, creatinine kinase, vitamin B1, B12, B6, folate, pyruvic acid, and thyroid-stimulating hormone were all unremarkable. Cerebral spinal fluid (CSF) studies revealed white blood cells (WBC) 10 cells/µL (neutrophil 83%, lymphocytes 10%), red blood cells (RBC) 9000 cells/µL, immunoglobulin G (IgG) within normal limits. CSF encephalitis, meningitis, and paraneoplastic panels were all negative. CSF cultures did not grow bacteria or fungus. CSF HIV, John Cunningham (JC) virus polymerase chain reaction (PCR), 14-3-3 protein were all negative. Magnetic resonance angiography (MRA) of the head and neck and MRI of cervical, thoracic, and lumbar spine were unrevealing.

Throughout her hospital stay, the patient remained confused, slow to respond, and unable to ambulate. She received supportive treatment and vitamin supplements, including coenzyme Q, vitamin C, and vitamin E, and underwent rigorous rehabilitation during her lengthy hospital stay. She was discharged home with no neurological symptoms. She failed to attend her follow-up appointment visit with her local neurologist. 

## Discussion

Toxic leukoencephalopathy (TLE) classically progresses through three stages over weeks to months. The first stage, primarily cerebellar, includes motor restlessness and ataxia with pseudobulbar speech. The second stage includes myoclonic jerks, choreoathetoid movements, and spastic paresis. Finally, approximately 25% of patients progress to the third stage, which includes akinetic mutism, extensor posturing, central pyrexia, and eventual death [3-11]. Toxic exposure from heroin-induced leukoencephalopathy often involves bilateral symmetric damage to cerebellar white matter, posterior cerebral white matter; typically, occipital lobe, posterior limb of the internal capsule, and cerebellar peduncles [12]. Brain MRI is not generally included as part of routine workup during psychiatric admission, but It should be considered in patients suspected to have heroin inhalation for possible TLE diagnosis [13]. In our cases, brain MRI showed the classical findings of TLE, including restricted diffusion in the white matter of both cerebral hemispheres with corresponding T2 and FLAIR hyperintensity. 

TLE can cause mild deficits, including inattention, agitation, altered mentation, memory retrieval deficits, and visual hallucination, which usually suggest a psychotic disorder. Most of these symptoms are disabling [14]. On the contrary to the cortical gray matter cognitive disorders such as Alzheimer's disease, TLE usually presents with preserved language, hemiparesis, sensory deficits, and vision loss are less likely to be seen. Neurobehavioral dysfunction reflects generalized involvement of white matter, best seen on T2-weight MRI scans [1].

The clinical features of the disease are expressed depending on the involved area of the brain. These features have varying severity ranging from inattention, forgetfulness, personality changes, and dysarthria to more severe symptoms such as ataxia, dementia, coma, and death [10]. Our two cases exhibited some components of first stage symptoms, but behavioral symptoms were thought to be dominant, prompting psychiatric admissions in both cases.

Toxic leukoencephalopathy (TLE) refers to progressive damage of the white matter of the brain, particularly myelin. TLE can be caused by drugs of abuse and their solvents, environmental toxins, and chemotherapeutic drugs [1]. Aluminum has been implicated as a potential causative contaminant toxin in the pathogenesis of toxic leukoencephalopathy [4]. Toxic leukoencephalopathy and neurologic symptoms associated with heroin abuse by inhalation were initially described in 1982 in the Netherlands [6]. Identification of acute-onset TLE after heroin inhalation has distinct implications for neuro-prognostication. In addition to heroin, other agents have been linked to TLE, such as toluene, ethanol, cocaine, methylenedioxymethamphetamine (MDMA or "ecstasy"). Heroin abuse has been identified in both of our cases. In our first case, heroin abuse was in the form of inhalation. The pathogenesis of TLE is not well understood. The prevailing hypothesis revolves around the assumption of heroin contaminated agents or combustion byproduct, causing the disease, although no agent has been identified yet [10]. Moreover, toluene has also been described in the literature as a solvent of abuse that can produce white matter toxicity [1].

According to Buxton et al., out of the 27 hospitalized cases of TLE, who were identified in British Columbia between December 2001 to August 2006, 13 cases were fatal - all had a three-year history of smoking heroin. The study suggested that dose-dependent exposure to chemicals mixed with heroin or byproduct combustion is possibly the cause of TLE [10]. Smoking heroin was identified in one of our patients, although the period was unknown. 

The prevailing neuropsychiatric symptoms such as personality and behavioral changes in both of our cases might have led to the misdiagnosis of TLE as a primary psychiatric disorder. While the diagnosis of TLE remains challenging due to the broad spectrum of clinical presentation, heroin-induced leukoencephalopathy should be suspected in patients with heroin use who presented mainly with acute or subacute neurobehavioral changes. That should be supported by a positive urine test for opioids along with characteristic neuroimaging findings and a history of heroin abuse [10]. 

Antioxidant agents including coenzyme Q, vitamin C, and vitamin E have been suggested to provide a varying degree of benefit in patients with toxic leukoencephalopathy knowing their safe profile adverse effect [4, 15- 18]. In our first case, antioxidant treatment did not improve symptoms, whereas in our second case, it resulted in remarkable improvement.

## Conclusions

Heroin-induced leukoencephalopathy is a rare but important neurological complication of heroin abuse. Neurologists should be cognizant of such complication in heroin abuse patients who present primarily with cognitive behavioral changes. The diagnosis is supported by a positive urine test for opioids along with characteristic neuroimaging findings and history of heroin abuse. 
